# Classification of brain states that predicts future performance in visual tasks based on co-integration analysis of EEG data

**DOI:** 10.1098/rsos.220621

**Published:** 2022-11-30

**Authors:** Marie Levakova, Jeppe Høy Christensen, Susanne Ditlevsen

**Affiliations:** ^1^ Department of Mathematical Sciences, University of Copenhagen, Universitetsparken 5, 2100 Copenhagen Ø, Denmark; ^2^ Eriksholm Research Centre, Rørtangvej 20, 3070 Snekkersten, Denmark

**Keywords:** EEG data, cointegration analysis, functional network

## Abstract

Electroencephalogram (EEG) is a popular tool for studying brain activity. Numerous statistical techniques exist to enhance understanding of the complex dynamics underlying the EEG recordings. Inferring the functional network connectivity between EEG channels is of interest, and non-parametric inference methods are typically applied. We propose a fully parametric model-based approach via cointegration analysis. It not only estimates the network but also provides further insight through cointegration vectors, which characterize equilibrium states, and the corresponding loadings, which describe the mechanism of how the EEG dynamics is drawn to the equilibrium. We outline the estimation procedure in the context of EEG data, which faces specific challenges compared with the common econometric problems, for which cointegration analysis was originally conceived. In particular, the dimension is higher, typically around 64; there is usually access to repeated trials; and the data are artificially linearly dependent through the normalization done in EEG recordings. Finally, we illustrate the method on EEG data from a visual task experiment and show how brain states identified via cointegration analysis can be utilized in further investigations of determinants playing roles in sensory identifications.

## 1. Introduction

An electroencephalogram (EEG) records the electrical activity of the brain in terms of differences in electrical potentials between two points by electrodes attached to the scalp. EEG recordings are an invaluable source of information about neuronal activity on a global level. Traditional statistical techniques focus on both the time and the frequency domain. In the time domain, stimulus-evoked responses at a single or at a few electrodes are typically assessed using event-related response methods [1], which average the time-locked EEG signal across repeated trials. Frequency domain techniques investigate the amplitude or phase of oscillatory activity in selected electrode(s) for specific frequency bands.

More recently, methods for assessing the functional network connectivity between electrodes have been developed [2,3]. The functional network connectivity describes how activity at each recording site is related to activity measured at other sites. These include computing Pearson correlations between the time series, estimating oscillatory coupling from, e.g. coherence or phase locking values within a time window [4], or estimating the causal influence from one electrode to others with Granger causality [5]. Thus, functional network connectivity methods assess the EEG activity of all the electrodes, adhering to the idea that the brain is a dynamical system and that electrodes should not be examined in isolation [6].

In this article, we propose cointegration analysis as a novel tool to infer functional network connectivity. The starting point is a multi-dimensional continuous stochastic differential equation model for the underlying process leading to a vector autoregressive (VAR) model of order 1 of the discretely observed EEG signal. This model considers all channels jointly and takes into account the autocorrelation in the data. Cointegration analysis is based on the VAR model. VAR models have previously been used for EEG data; for example, autoregressive models were used to calculate a direct transfer function [7], partial directed coherence [8], or in classification algorithms [9]. However, the standard VAR model is assumed stationary, which is not the case for EEG data during stimulation, and the phenomenon of spurious regression may arise [10].

Cointegration analysis is able to address the four following aspects in EEG network analysis. First, most of the usual methods are non-parametric, whereas our approach is fully parametric. While a clear benefit of non-parametric and semi-parametric procedures is that they are (nearly) free from limiting assumptions and flexibly applicable under most circumstances, a fully model-based parametric approach offers a framework that can answer more specific scientific questions. Moreover, interpretation of parameters is more transparent, and estimation variance is reduced in the parametric setting.

Second, the most common methods study connectivity in the frequency domain, which requires observation intervals that are long enough to extract the most prominent cyclical components. However, the period of the alpha rhythm is between 80 and 125 ms. Here, we estimate the functional network connectivity during a short stimulation period lasting only between 20 and 110 ms, and we determine the brain state from only 100 ms before stimulus onset. Thus, inference in the frequency domain is not an option.

Third, a crucial assumption in standard time series analysis and a common assumption in general is that EEG data are stationary. The natural physical limitations of EEG data do not allow for persistent trends. However, there will be temporary deviations due to experimental inputs. This non-stationarity has implications for the statistical analysis, such as spurious regression [10]. Finally, networks are typically inferred among a limited number of channels or among a few larger brain areas. Our aim is to upscale the cointegration methodology so that all EEG channels (64 in our case) are included in the network.

Cointegration analysis was originally developed with econometrics applications in mind [11]. Recently, it has been applied to climate research [12], phase-coupled oscillating systems in physics [13] and to low-dimensional systems of coupled oscillators in neuroscience [14]. The idea of cointegration analysis is to discern which part of the data can be attributed to stochastic trends and which part stems from linear equilibrium relationships, termed cointegration relationships. This is particularly relevant for non-stationary data, which nevertheless exhibit some kind of stability or structure in the overall system. Such dynamics are observed in many biological systems, e.g. in processes in the brain measured through EEG, which we focus on here. The cointegration relationships then represent the functional network connectivity between electrodes in the EEG.

The estimated parameters of the cointegration model are relevant for interpreting the EEG: the cointegration rank gives the number of independent cointegration relationships related to the global connectivity network and the number of independent stochastic trends; the cointegration matrix contains coefficients of cointegration relationships (i.e. electrode connectivity strengths); the loading matrix describes how the underlying system (i.e. the brain) reacts to deviations from the cointegration relationships. Most importantly, the product of the loading and the cointegration matrices describes the functional network structure. The structure might change over time, which reflects for example how brain states change in response to changing tasks.

We apply the cointegration analysis to EEG data obtained from two human participants performing a simple visual identification task. In each trial, the participant was asked to report the orientation of a briefly presented Landolt ring. Exogenous factors such as the presentation time and stimulus luminance are the typical factors that predict performance in the task; however, pre-stimulus EEG activity has recently been shown also to have predictive value in similar tasks [15]. That is, the brain activity prior to the onset of the visual stimulus affects how well the stimulus is perceived, either from oscillatory activity in the visual cortex timed to the onset of sensory input [16,17] or from spontaneous activity [18]. We show that cointegration analysis can be used to investigate this further while giving an account of the functional networks involved. We then test if the pre-stimulus brain state estimated from a time interval as short as 100 ms predicts performance on the visual identification task.

## 2. Cointegration methodology

The vector $x_{t}={\left(x_{1t},x_{2t},\ldots ,x_{\;pt}\right)}^{\prime }\in R^{p}$ represents the EEG signals recorded at time *t*, *p* is the number of electrodes and ′ denotes the transpose. If not stated otherwise, then *p* = 64. The data are recorded at time points *t*_0_ < *t*_1_ < … < *t*_*n*_ < … < *t*_*N*_. We write *x*_*n*_ for $x_{t_{n}}$, and *x*_0:*N*_ denotes the set of observations {*x*_*n*_ : *n* = 0, …, *N*}. For *M* repeated trials, $x_{n}^{\left(m\right)}$ is the vector of recordings at time *t*_*n*_ in trial *m* and *x*^(1:*M*)^ denotes the set of all observations in all trials $\left\{x_{n}^{\left(m\right)}\;:\;n=0,\ldots ,N_{m},m=1,\ldots ,M\right\}$, where *N*_*m*_ is the number of observations in trial *m*. A *p*-dimensional column vector of zeros is denoted by **0**_*p*_, a *p*-dimensional column vector of ones is denoted by **1**_*p*_ and **I**_*p*_ is the *p* × *p* unit matrix. The trace of a generic square matrix *A* is tr(*A*), *A*_*i*·_ represents the *i*th row of the matrix *A* and *A*_·*i*_ is the *i*th column of *A*. The *l*_2_-norm of a vector $v\in R^{p}$ is ${\left\|v\right\|}_{2}=\sqrt{\sum_{i=1}^{p}v_{i}^{2}}$ and the *l*_1_-norm is ${\left\|v\right\|}_{1}=\sum_{i=1}^{p}|v_{i}|$. Finally, a hat marks an estimate, i.e. $\hat{\mu }$ is an estimate of parameter *μ*.

### 2.1. Model

Assume that the EEG signals evolve according to an Ornstein–Uhlenbeck process [19,20],

2.1 $$d\;x_{t}=P(x_{t}-m)\;dt+D\;dW_{t},$$

where *m* is a *p*-dimensional mean vector, *P* is a *p* × *p* matrix, *D* is a *p* × *d* matrix with *d* ≤ *p* such that *DD*′ is positive semidefinite and *W* is a *d*-dimensional Brownian motion. To ensure that the process generated by (2.1) is recurrent, the eigenvalues of *P* must all have negative real parts (positive recurrent) or some of them be equal to 0 (null recurrent). The matrix *P* can be factorized as *P* = *ab*′, where $a,b\in R^{\;p\times r}$ and *r* ≤ *p*. We assume furthermore that |*b*′*a*| ≠ 0 and all eigenvalues of *b*′*a* have negative real parts.

If the process is observed at equidistant time points *t*_1_, …, *t*_*N*_ with timestep Δ = *t*_*n*_ − *t*_*n*−1_, the observations *x*_*n*_, *n* = 1, …, *N* satisfy the VAR model

2.2 $$x_{n}=\mu +Ax_{n-1}+\varepsilon_{n},\;\varepsilon_{n}∼N_{p}(0_{p},\Sigma ),\;n=1,\ldots ,N,$$

where *A* = e^*P*Δ^, *μ* = (**I**_*p*_ − *A*)*m* and $\Sigma =\int_{0}^{\Delta }e^{uP}DD^{\prime }e^{uP^{\prime }}\;du$. It is more convenient to write the VAR process as a *vector error–correction model* (VECM),

2.3 $$\Delta x_{n}=x_{n}-x_{n-1}=\mu +\Pi x_{n-1}+\varepsilon_{n},\;n=1,\ldots ,N,$$

with $\Pi =A-I_{p}$. The assumptions imposed on matrices *a* and *b* imply that all eigenvalues of *A* have modulus less than 1 or are equal to 1 [21].

The rank of Π in (2.3), r=rank(Π), has fundamental implications for the properties of the system, directly linking to the properties of the original system (2.1), since rank(Π)=rank(P) [21].

— If *r* = 0, ${\left\{x_{n}\right\}}_{n=1}^{N}$ is a set of *p* random walks, as $\Delta x_{n}=\varepsilon_{n}$.
— If *r* = *p*, all eigenvalues of *A* have modulus less than 1, ${\left\{x_{n}\right\}}_{n=1}^{N}$ is asymptotically stationary and contains neither a stochastic trend nor a linear trend [22].
— If 0 < *r* < *p*, ${\left\{x_{n}\right\}}_{n=1}^{N}$ is driven by exactly *p* − *r* independent stochastic trends, and there exist *r* linear combinations of the vector process ${\left\{x_{n}\right\}}_{n=1}^{N}$ that yield an asymptotically stationary one-dimensional process. Linear trends due to the drift *μ* are possible too.

The third case is termed cointegration and will be assumed from now on. If 0 < *r* < *p*, we can find matrices $\alpha ,\beta \in R^{\;p\times r}$ such that $\Pi =\alpha \beta^{\prime }$. The matrices *α* and *β* are not unique, one can take an arbitrary invertible matrix $Q\in R^{r\times r}$ and find another decomposition $\Pi =(\alpha Q)(Q^{-1}\beta^{\prime })=\alpha^{*}\beta^{{*}^{\prime }}$. However, the subspaces spanned by the columns of *α* and *β*, *sp*(*α*) and *sp*(*β*), are unique [21].

Matrix Π has a straightforward interpretation. An element $\Pi_{ij}$ quantifies the influence of channel *j* on the change in channel *i* in the following time point. The matrix Π thus defines a functional network among the channels 1, …, *p*. The matrix *β* is called the *cointegration matrix*, since the columns of *β* contain *cointegration vectors*. A cointegration vector $v\in R^{p}$ is a vector such that *v*′*x*_*n*_ is an asymptotically stationary univariate process. Thus, a linear combination given by *v* represents an equilibrium, although single EEG signals can be subject to stochastic trends. Cointegration vectors form a linear subspace, meaning that any linear combination of two distinct cointegration vectors is also a cointegration vector. The columns of *β* are one possible basis of this subspace.

The matrix *α* is called the *loading matrix*. It describes the correcting mechanism ensuring that *β*′*x*_*n*_ is always pushed to the long-term mean $E(\beta^{\prime }x_{\infty })\;:=\lim_{n\to \infty }E(\beta^{\prime }x_{n})$. Rewrite the VECM model (2.3) as follows:

2.4 $$\Delta x_{n}=\underset{\text{a constant vector}}{\underbrace{\mu +\alpha E(\beta^{\prime }x_{\infty })}}+\;\alpha \underset{\text{disequilibrium error}}{\underbrace{\left[\beta^{\prime }x_{n-1}-E(\beta^{\prime }x_{\infty })\right]}}+\varepsilon_{n}.$$

The matrix *α* thus reveals the rate at which the system reacts to the deviations of the cointegration relationships *β*′*x*_*n*−1_ from the asymptotic mean $E(\beta^{\prime }x_{\infty })$ to keep the stationary cointegration relationships satisfied in the long term.

### 2.2. Estimation procedure

We briefly review the most common estimation procedure termed the *Johansen procedure* [23]. We will show the non-uniqueness of some of the estimated parameters and how a particular choice of an estimate affects the rest, and address particular issues arising for EEG datasets.

#### 2.2.1. Single trial

The Johansen procedure is based on the maximum likelihood method. Assuming $\varepsilon_{n}∼N(0,\Sigma )$ and centring Δ*x*_*n*_ and *x*_*n*_ as follows:

2.5 $$z_{0n}=\Delta x_{n}-\frac{1}{N}\sum_{n=1}^{N}\Delta x_{n};\;z_{1n}=x_{n-1}-\frac{1}{N}\sum_{n=1}^{N}x_{n-1},$$

the log-likelihood becomes

2.6 $$\ell (\alpha ,\beta ,\Sigma ;z_{0,1:N},z_{1,1:N})\propto -\frac{N}{2}\log|\Sigma |-\frac{1}{2}\sum_{n=1}^{N}{\left(z_{0n}-\alpha \beta^{\prime }z_{1n}\right)}^{\prime }\Sigma^{-1}(z_{0n}-\alpha \beta^{\prime }z_{1n}).$$

The parameter estimates can be expressed in terms of sufficient statistics *S*_00_, *S*_01_ and *S*_11_, which are obtained as follows:

2.7 $$S_{ij}=N^{-1}\sum_{n=1}^{N}z_{in}z_{\;jn}^{\prime },\;i,j\in \{1,2\}.$$

The estimation procedure has the following consecutive steps:

(i) Estimate the cointegration rank *r*
(ii) Estimate the cointegration matrix *β*
(iii) Estimate the loading matrix *α*
(iv) Estimate the covariance matrix Σ and the drift *μ*

We explain the steps (ii)–(iv) first and discuss the topic of the cointegration rank determination in more detail in §2.2.5.

*Estimation of β.* For a given cointegration rank *r*, the task is to estimate Π subject to rank(Π)=r, which is a problem of *reduced rank regression* [24]. The columns of *β* can be found as the eigenvectors *v*_1_, …, *v*_*r*_ corresponding to the *r* largest eigenvalues *λ*_*i*_ of the eigenvalue problem,

2.8 $$\lambda_{i}S_{11}v_{i}=S_{01}^{\prime }S_{00}^{-1}S_{01}v_{i},\;i=1,\ldots ,p,$$

which are normalized so that

2.9 $$v_{i}^{\prime }S_{11}v_{j}=\left\{ \begin{array}{cc} 1\; & \text{for }i=j,\;\\ 0\; & \text{for }i\neq j.\; \end{array}\right.$$

Since any linear combination of the cointegration vectors yields a cointegration vector, any basis of $sp(\hat{\beta })$ can serve as $\hat{\beta }$.

*Estimation of *α*.* For fixed $\hat{\beta }$, we construct new stationary covariates ${\hat{\beta }}^{\prime }z_{1n}=u_{n}\in R^{r}$ for each *n* ∈ {1, …, *N*} and turn the VECM model (2.3) into a standard linear regression model,

2.10 $$z_{0n}=\alpha u_{n}+\varepsilon_{n},$$

with no restrictions on the rank. The maximum likelihood estimator (MLE) as well as the standard least squares estimator are expressed as follows:

2.11 $$\hat{\alpha }=S_{01}\hat{\beta }{\left({\hat{\beta }}^{\prime }S_{11}\hat{\beta }\right)}^{-1}.$$

The chosen form of $\hat{\beta }$ affects $\hat{\alpha }$; however, $sp(\hat{\alpha })$ is invariant. Furthermore, $\hat{\Pi }=\hat{\alpha }{\hat{\beta }}^{\prime }$ is always unique. The estimation procedure gives some flexibility for choosing $\hat{\beta }$, but not for $\hat{\alpha }$. There exist also procedures where $\hat{\alpha }$ is identified first with certain degree of freedom, and then, the estimator of *β* given $\hat{\alpha }$ is unique [25].

*Estimation of Σ and μ*. The closed-form expressions of the MLE of Σ and *μ* for fixed $\hat{\beta }$ are as follows:

2.12 $$\hat{\Sigma }=S_{00}-S_{01}\hat{\beta }{\left({\hat{\beta }}^{\prime }S_{11}\hat{\beta }\right)}^{-1}{\hat{\beta }}^{\prime }S_{01}^{\prime }$$

and

2.13 $$\hat{\mu }=\frac{1}{N}\sum_{i=1}^{N}(\Delta x_{n}-\hat{\alpha }{\hat{\beta }}^{\prime }x_{n-1}).$$

#### 2.2.2. Repeated trials

Cointegration analysis has mainly been used in econometrics, where processes of interest cannot be repeated, and statistical inference is based on single time series observed typically over a long period of time. The data in experimental neurobiology often differ in two aspects: processes evolve over a short time interval and only few observations can be made, and running the same experiment under controlled conditions repeatedly is not a problem. Here, we show how cointegration analysis can be performed with data from repeated trials.

Assume the process (2.3) was observed in *M* experimental trials with *N*_*m*_ observations in trial *m*. The log-likelihood becomes

2.14 $$\begin{array}{rl} & \ell (\mu ,\alpha ,\beta ,\Sigma ;x^{\left(1:M\right)})\propto -\frac{\log|\Sigma |}{2}\sum_{m=1}^{M}N_{m}\\ & \;-\frac{1}{2}\sum_{m=1}^{M}\sum_{n=1}^{N_{m}}{\left(\Delta x_{n}^{\left(m\right)}-\alpha \beta^{\prime }x_{n-1}^{\left(m\right)}-\mu \right)}^{\prime }\Sigma^{-1}\left(\Delta x_{n}^{\left(m\right)}-\alpha \beta^{\prime }x_{n-1}^{\left(m\right)}-\mu \right), \end{array}$$

and the only change is that now we also sum over trials *m*. The sufficient statistics $S_{00}^{M}$, $S_{01}^{M}$ and $S_{11}^{M}$ are as follows:

2.15 $$S_{ij}^{M}={\left(\sum_{m=1}^{M}N_{m}\right)}^{-1}\sum_{m=1}^{M}\sum_{n=1}^{N_{m}}z_{in}^{\left(m\right)}z_{\;jn}^{{\left(m\right)}^{\prime }},$$

where

2.16 $$z_{0n}^{\left(m\right)}=\Delta x_{n}^{\left(m\right)}-{\left(\sum_{m=1}^{M}N_{m}\right)}^{-1}\sum_{m=1}^{M}\sum_{i=1}^{N_{m}}\Delta x_{n}^{\left(m\right)}$$

and

2.17 $$z_{1n}^{\left(m\right)}=x_{n-1}^{\left(m\right)}-{\left(\sum_{m=1}^{M}N_{m}\right)}^{-1}\sum_{m=1}^{M}\sum_{i=1}^{N_{m}}x_{n-1}^{\left(m\right)}.$$

Analogously to a single trial, $\hat{\beta }$ is constructed from *r* eigenvectors *v*_*i*_, *i* = 1, …, *r*, corresponding to the *r* largest eigenvalues of the problem

2.18 $$\lambda_{i}S_{11}^{M}v_{i}=S_{01}^{M^{\prime }}{\left(S_{00}^{M}\right)}^{-1}S_{01}^{M}v_{i},\;i=1,\ldots ,p,$$

and the estimators of *α*, Σ and *μ* are obtained as follows:

2.19 $$\hat{\alpha }=S_{01}^{M}\hat{\beta }{\left({\hat{\beta }}^{\prime }S_{11}^{M}\hat{\beta }\right)}^{-1},$$

2.20 $$\hat{\Sigma }=S_{00}^{M}-S_{01}^{M}\hat{\beta }{\left({\hat{\beta }}^{\prime }S_{11}^{M}\hat{\beta }\right)}^{-1}{\hat{\beta }}^{\prime }S_{01}^{M^{\prime }}$$

2.21 $$\mathrm{and}\;\;\;\;\;\;\hat{\mu }={\left(\sum_{m=1}^{M}N_{m}\right)}^{-1}\sum_{m=1}^{M}\sum_{i=1}^{N_{m}}\left(\Delta x_{n}^{\left(m\right)}-\hat{\alpha }{\hat{\beta }}^{\prime }x_{n-1}^{\left(m\right)}\right).$$

#### 2.2.3. Dealing with the reference level in electroencephalogram measurements

EEG measures differences in electrical potentials between two points. Thus, the signal at any channel is the difference to some recording site. This recording site is the baseline electrode, which is, however, prone to pick up electrical noise that does not reach the other electrodes. Consequently, the voltage differences between baseline and other electrodes are also affected by this noise.

To eliminate this noise, systems for recording EEG usually re-reference EEG signals with respect to another reference level that is chosen from the EEG channels. The signals at the other EEG channels are expressed as the differences in electrical potential to this reference instead of the baseline. This cancels out the noise stemming from the baseline circuit; however, it introduces linear dependence between the recordings and causes the cointegration model to be overparametrized. We will illustrate how this can be handled, for the case of a *common average reference*, which is a frequent choice of the reference level.

The new reference is the average electrical activity measured across all channels and re-referencing is achieved by subtracting the average from each channel. The electrical activity across all channels therefore sums to zero at each time point,

2.22 $$\sum_{i=1}^{p}x_{in}=0,\;\forall n\in \{1,\ldots ,N\},$$

and the signal from one of the channels can always be derived from the other *p* − 1 channels.

Assume that the *p*th channel is excluded when the cointegration matrix is estimated. The new dataset consists of (*p* − 1)-dimensional observations $x_{n}^{\left(p-1\right)}={\left(x_{1n},\ldots ,x_{\;p-1,n}\right)}^{\prime }$, and the model is

2.23 $$\Delta x_{n}^{\left(p-1\right)}=\mu^{\left(p-1\right)}+\alpha^{\left(p-1\right)}\beta^{{\left(p-1\right)}^{\prime }}x_{n-1}^{\left(p-1\right)}+\varepsilon_{n}^{\left(p-1\right)},$$

where $\alpha^{\left(p-1\right)},\beta^{\left(p-1\right)}\in R^{(p-1)\times r}$, $\mu^{\left(p-1\right)}\in R^{\;p-1}$ and $\varepsilon_{n}^{\left(p-1\right)}∼N_{\;p-1}(0,\Sigma^{\left(p-1\right)})$. A cointegration relationship in the (*p* − 1)-dimensional model given by the *j*th column of *β*^(*p*−1)^ can be written as a linear combination of the full *p*-dimensional vector *x*_*n*_ using (2.22) as follows:

2.24 $$\beta_{\cdot j}^{{\left(p-1\right)}^{\prime }}x_{n}^{\left(p-1\right)}={\left(\beta_{\cdot j}^{\left(p-1\right)}+c_{j}1_{\;p-1}\right)}^{\prime }x_{n}^{\left(p-1\right)}+c_{j}x_{\;pn},\;j\in \{1,\ldots ,r\},$$

where $c_{j}\in R$ is an arbitrary constant. If *β*^(*p*−1)^ is chosen so that the normalization condition $\beta^{{\left(p-1\right)}^{\prime }}S_{11}^{\left(p-1\right)}\beta^{\left(p-1\right)}=I_{r}$ holds, the *p*-dimensional cointegration matrix *β* with columns $\beta_{\cdot j}={\left(\beta_{\cdot j}^{{\left(p-1\right)}^{\prime }}+c_{j}1_{\;p-1}^{\prime },c_{j}\right)}^{\prime }$ satisfies the analogous *p*-dimensional condition *β*′*S*_11_*β* = **I**_*r*_ for any $c_{j}\in R$, *j* ∈ {1, …, *r*}. This is because the common mode reference implies *S*_11_**1**_*p*_ = **0**_*p*_. In the analysis in §3, we use $c_{j}=-(1/\;p)\sum_{i=1}^{\;p-1}\beta_{ij}^{\left(p-1\right)}$ to minimize the norm ‖*β*_·*j*_‖_2_.

The matrix $\hat{\alpha }$ is obtained from the full *p*-dimensional model using formula (2.19). Note that $\hat{\Pi }=\hat{\alpha }{\hat{\beta }}^{\prime }$ in the full *p*-dimensional model is not invariant with respect to the choice of the excluded channel and with respect to the choice of constants *c*_*j*_, *j* = 1, …, *r*. However, the product $\hat{\Pi }x_{n-1}$ is invariant. Specifically, $\hat{\Pi }x_{n-1}$ remains unchanged if an arbitrary constant *d*_*i*_ is added to all elements in a row ${\hat{\Pi }}_{i\cdot }$, *i* ∈ {1, …, *p*}.

#### 2.2.4. Regularization

As the dimension *p* is typically high for EEG data, it is beneficial to impose regularization to obtain robust estimators. Several approaches have been suggested: imposing lasso penalty directly on Π [26], on *β* [25] or regularizing certain decompositions of Π [27,28]. Here, we penalize *α*. The reasons for choosing penalization of *α* over other possible methods are as follows: (i) easy computation that can be done with standard software packages, (ii) no bias is introduced into the estimated cointegration space $sp(\hat{\beta })$, and (iii) better performance over a range of error measures compared with other penalization methods [29].

First, the cointegration matrix *β* is estimated by the Johansen procedure, yielding $\hat{\beta }$. Then, we find $\hat{\alpha }$ by minimizing the sum of squared errors with elastic net penalty [30]

2.25 $$\begin{array}{rl} \hat{\alpha } & =\arg\min_{\alpha \in R^{\;p\times r}}\frac{1}{2N}\sum_{m=1}^{M}\sum_{n=1}^{N_{m}}{\left(z_{0n}^{m}-\alpha {\hat{\beta }}^{\prime }z_{1n}^{\left(m\right)}\right)}^{\prime }\left(z_{0n}^{m}-\alpha {\hat{\beta }}^{\prime }z_{1n}^{\left(m\right)}\right)\\ & \;+\gamma \left[\frac{1-\omega }{2}\sum_{i=1}^{p}{\left\|\alpha_{i\cdot }\right\|}_{2}^{2}+\omega \sum_{i=1}^{p}{\left\|\alpha_{i\cdot }\right\|}_{1}\right], \end{array}$$

where *γ* ≥ 0 is a tuning parameter governing the overall amount of penalization and *ω* ∈ [0, 1] controls the proportion of the ridge penalty ${\left\|\alpha_{i\cdot }\right\|}_{2}^{2}/2$ and the lasso penalty ‖*α*_*i*·_‖_1_. The lasso penalty pushes the elements of $\hat{\alpha }$ to become exact zeros and thus a sparse representation, allowing for a more meaningful interpretation. The ridge penalty weakens the impact of potential correlation between the predictors ${\hat{\beta }}^{\prime }z_{1n}^{\left(m\right)}$. A sparse estimate $\hat{\alpha }$ does not imply that $\hat{\Pi }=\hat{\alpha }{\hat{\beta }}^{\prime }$ is sparse.

#### 2.2.5. Determination of the cointegration rank

In standard low-dimensional problems, the typical procedure to determine *r* is based on likelihood ratio tests that are applied sequentially over a range of possible cointegration ranks. The likelihood ratio test can be in two forms. The null hypothesis is the same, *H*_0_ : *r* ≤ *r*_0_, while the alternative is either *H*_*a*_ : *r* ≤ *p* (trace test) or *H*_*a*_ : *r* ≤ *r*_0_ + 1 (maximum eigenvalue test). The test statistics depend only on eigenvalues *λ*_*i*_, *i* = 1, …, *p*, of the eigenvalue problem (2.18). Assuming that the eigenvalues are in a descending order, *λ*_1_ ≥ … ≥ *λ*_*p*_, the test statistics are as follows:

— trace test:
  2.26 $$-2[\ell (r_{0})-\ell (p)]=-\left(\sum_{m=1}^{M}N_{m}\right)\sum_{i=r_{0}+1}^{p}\log(1-\lambda_{i}),$$
— maximum eigenvalue test:
  2.27 $$-2[\ell (r_{0})-\ell (r_{0}+1)]=-\left(\sum_{m=1}^{M}N_{m}\right)\log(1-\lambda_{r_{0}+1}).$$

The sequential testing starts with setting *r*_0_ = 0. If *H*_0_ is rejected, *r*_0_ is increased by one, and the test is repeated until acceptance.

This way of determining the rank is in general not applicable for EEG data due to their high dimension. The trace and the maximum eigenvalue test statistics do not follow any standard distribution, and their critical values depend on *p* and need to be calculated numerically. Currently, critical values are available for dimension *p* ≤ 11. This can be overcome by bootstrap methods, but the required computer time makes them out of reach.

*Eigenvalues*. The eigenvalues of (2.18) are approximate indicators of the cointegration rank. If a cointegration relationship *v*_*i*_ exists, the corresponding eigenvalue *λ*_*i*_ is expected to be significantly larger than zero. A rough insight can therefore be gained from a scree plot, where ordered eigenvalues are plotted against the rank.

Bunea *et al.* [31] proposed a *rank selection criterion* that uses eigenvalues of $S_{01}^{M}{\left(S_{11}^{M}\right)}^{-1}S_{10}^{M}$ and identifies the cointegration rank as the number of eigenvalues larger than or equal to a threshold *θ*,

2.28 $$\theta =\frac{2(p+q)}{p(\sum_{m=1}^{M}N_{m}-q)}\sum_{m=1}^{M}\sum_{n=1}^{N_{m}}{\left(z_{0n}^{\left(m\right)}-{\hat{\Pi }}_{p}z_{1n}^{\left(m\right)}\right)}^{\prime }\left(z_{0n}^{\left(m\right)}-{\hat{\Pi }}_{p}z_{1n}^{\left(m\right)}\right),$$

where ${\hat{\Pi }}_{p}$ is the MLE of Π assuming full rank *p* and *q* is the rank of the matrix of predictors $Z_{1}={\left(z_{11},\ldots ,z_{1N_{M}}\right)}^{\prime }$. The cointegration rank selected by the rank selection criterion is equal to the rank of $\hat{\Pi }$ estimated by a penalized least squares estimator of the form

2.29 $$\hat{\Pi }=\arg\min_{\Pi }\left[\sum_{m=1}^{M}\sum_{n=1}^{N_{m}}{\left(z_{0n}^{\left(m\right)}-\Pi z_{1n}^{\left(m\right)}\right)}^{\prime }\left(z_{0n}^{\left(m\right)}-\Pi z_{1n}^{\left(m\right)}\right)+\theta \;\mathrm{rank}(\Pi )\right].$$

*Matrix angle*. We use the following generalized version of the vector angle for quantifying the closeness of subspaces spanned by two matrices *U* and *V*,

2.30 $$\Theta (U,V)=\arccos\left(\frac{{\left\langle U,V\right\rangle }_{F}}{\sqrt{{\left\langle U,U\right\rangle }_{F}{\left\langle V,V\right\rangle }_{F}}}\right),$$

and we call it the angle between matrices $U,V\in R^{\;p\times p}$. It uses the Frobenius inner product 〈*U*, *V*〉_*F*_ = tr(*U*′*V*). For *U* = 0 or *V* = 0, we set Θ(U,V)=π/2.

The angle $\Theta (\hat{\Pi },\Pi )$ between the estimate $\hat{\Pi }$ and the true Π depends on how close the estimation rank is to the true rank. When the estimation rank is much smaller than the true rank, the estimate $\hat{\Pi }$ is far from the truth and the angle $\Theta (\hat{\Pi },\Pi )$ can be as high as *π*/2. When the estimation rank approaches the true rank, the angle $\Theta (\hat{\Pi },\Pi )$ tends to decrease. However, when the estimation rank increases beyond the true rank, the angle does not decrease further but fluctuates around a constant level due to sampling error.

Ideally, we could plot $\Theta ({\hat{\Pi }}_{r},\Pi )$ as a function of the estimation rank and inspect if the curve has a kink separating the decreasing and constant segments. However, the true Π is unknown. A naive solution is to replace Π with the estimate under full estimation rank ${\hat{\Pi }}_{p}$, but this produces a curve that decreases to zero, becoming exactly 0 for rank *p*, since the matrices are then equal.

Instead we replace the true Π with several estimates ${\hat{\Pi }}_{p}^{\left(-k\right)}$. We split the original dataset into *K* folds and estimate Π from data with the *k*th fold excluded, assuming full rank. Then we calculate estimates $\Pi_{r}^{\left(k\right)}$ under ranks *r* ∈ {0, …, *p*}, using only data from fold *k*. The plots of $\Theta ({\hat{\Pi }}_{r}^{\left(k\right)},{\hat{\Pi }}_{p}^{\left(-k\right)})$ against *r* are useful for two reasons. First, $\Theta ({\hat{\Pi }}_{r}^{\left(k\right)},{\hat{\Pi }}_{p}^{\left(-k\right)})$ does not go to zero due to sampling error. Second, $\Theta ({\hat{\Pi }}_{r}^{\left(k\right)},{\hat{\Pi }}_{p}^{\left(-k\right)})$ tends to have little variance for ranks lower than the true one, but can vary a lot for ranks higher than the true one.

*Cross-validation*. Another option is to compare the prediction error of the cointegration model with different ranks when applied to independent data through cross-validation, using *K* folds as mentioned earlier. We use the following two criteria:

1. *Mean squared error (MSE) of prediction:*
  2.31 $$\begin{array}{rl} & \mathrm{MSE}(r)\\ & =\frac{1}{K}\sum_{k=1}^{K}\left[\frac{1}{N_{k}}\sum_{n\in I_{k}}{\left(\Delta x_{n}^{\left(k\right)}-{\hat{\Pi }}_{r}^{\left(-k\right)}x_{n-1}^{\left(k\right)}-{\hat{\mu }}^{\left(-k\right)}\right)}^{\prime }\left(\Delta x_{n}^{\left(k\right)}-{\hat{\Pi }}_{r}^{\left(-k\right)}x_{n-1}^{\left(k\right)}-{\hat{\mu }}^{\left(-k\right)}\right)\right]. \end{array}$$
2. *Average cross-validated log-likelihood:*
  2.32 $$\begin{array}{rl} \ell (r) & =\frac{1}{K}\sum_{k=1}^{K}\{-\frac{1}{2N_{k}}\sum_{n\in I_{k}}[\log|{\hat{\Sigma }}_{r}^{\left(-k\right)}|\\ & \;+{\left(\Delta x_{n}^{\left(k\right)}-{\hat{\Pi }}_{r}^{\left(-k\right)}x_{n-1}^{\left(k\right)}-{\hat{\mu }}^{\left(-k\right)}\right)}^{\prime }{\left({\hat{\Sigma }}_{r}^{\left(-k\right)}\right)}^{-1}\left(\Delta x_{n}^{\left(k\right)}-{\hat{\Pi }}_{r}^{\left(-k\right)}x_{n-1}^{\left(k\right)}-{\hat{\mu }}^{\left(-k\right)}\right)]\}, \end{array}$$

where $\left\{x_{n}^{\left(k\right)}:n\in I_{k}\right\}$ are the observations in the *k*th fold specified by an index set *I*_*k*_, *N*_*k*_ is the number of observations in the *k*th fold and ${\hat{\mu }}^{\left(-k\right)}$, ${\hat{\Pi }}^{\left(-k\right)}$ and ${\hat{\Sigma }}^{\left(-k\right)}$ are estimates obtained when the *k*th fold is left out.

#### 2.2.6. Test of structural difference between two cointegrated networks

A natural question is whether the network is the same under two different experimental set-ups A and B, or whether the experimental conditions impose a structural difference of the network. We use the *Chow test*, with the null hypothesis being that the data from both subsets follow the same model with identical parameters *μ*_0_ and $\Pi_{0}$,

2.33 $$H_{0}:\;\Delta x_{n}^{\left(k\right)}=\mu_{0}+\Pi_{0}x_{n-1}^{\left(k\right)}+\varepsilon_{n}^{\left(k\right)},\;n=1,\ldots ,N_{k},\;k\in \{A,B\},$$

while the alternative is that each subset is governed by parameters $\left(\mu_{A},\Pi_{A}\right)$ or $\left(\mu_{B},\Pi_{B}\right)$, as follows:

2.34 $$H_{a}:\;\Delta x_{n}^{\left(k\right)}=\mu_{k}+\Pi_{k}x_{n-1}^{\left(k\right)}+\varepsilon_{n}^{\left(k\right)},\;n=1,\ldots ,N_{k},\;k\in \{A,B\}.$$

The test statistic is the usual likelihood ratio test statistic,

2.35 $$Q(H_{0}|H_{a})=-2\left[\ell \left(\mu_{0},\Pi_{0};x^{\left(A,B\right)}\right)-\ell \left(\mu_{A},\Pi_{A};x^{\left(A\right)}\right)-\ell \left(\mu_{B},\Pi_{B};x^{\left(B\right)}\right)\right]$$

and the asymptotic distribution is *χ*^2^ with *p*(2*r* + 1) − *r*^2^ degrees of freedom [22,32], given that the VAR model with lag order 1 is valid.

When the hypothesis is rejected, it is of interest to identify which elements are different. A range of likelihood ratio tests of constant loading matrix *α* or constant cointegration matrix *β* under either constant or variable cointegration rank can be found in the literature [32].

## 3. Application to electroencephalogram recordings from a visual task experiment

**Figure 1.**
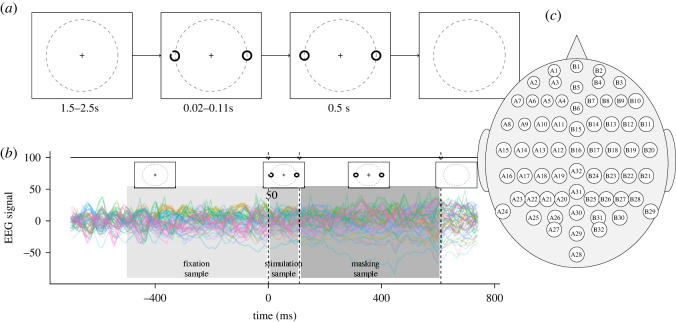
Set-up of the experiment. (*a*) Time course of the visual task. It starts with 1.5–2.5 s of fixation on the centrally located cross. Then two rings are shown on the sides, one with a gap (a Landolt C). After 0.02–0.11 s, the gap is masked. The masks are shown for 0.5 s, then a blank screen appears. (*b*) Example of one trial of EEG recordings, with three samples: fixation (the last 500 ms of the fixation period), stimulation (the whole duration of the stimulation period) and masking (500 ms period after the stimulation). (*c*) A 64-channel BioSemi EEG set-up on the scalp.

We applied the cointegration methodology to EEG recordings obtained during a visual identification experiment (figure 1), during which two participants were given the following task. First, the participant fixated on a centrally located fixation cross on a screen. The fixation lasted between 1.5 and 2.5 s randomly chosen. Then two rings appeared, one on each side of the fixation cross. Either the left or the right ring was a *Landolt C*, i.e. it had a gap at a certain orientation. The orientation was chosen from eight possible angles evenly spaced from 0 to 315 degrees in intervals of 45 degrees. The luminance contrast of the rings was either 6.5% or 28%. The duration of the visual stimulus was 20, 40, 70 or 110 ms. The position, orientation, contrast and duration were selected randomly with equal probabilities. The gap in the Landolt C was then masked for the 500 ms, followed by a blank screen. The participants had to report the orientation of the gap in the Landolt C. We classify participants’ answers into two categories: correct and incorrect.

EEG signals were recorded throughout the whole experimental session. They were obtained with 64 channel BioSemi EEG recording device (figure 1*c*) with 1024 Hz sampling (*post hoc* downsampled to 256 Hz). The data were cleaned with automatic methods from EEGlab in Matlab and re-referenced using a common mode reference. Excessive kurtosis was detected in four channels for Participant 1 (A2, B1, B2 and B12) and in three channels for Participant 2 (B29, B30 and B31); these channels were excluded from further analysis. Each participant completed 768 trials (8 orientations×2 contrasts×4 durations×12 repetitions). After cleaning the data, there were 609 trials for Participant 1 and 587 trials for Participant 2. The two study participants were healthy, young female university students (20 and 21 years old).

The statistical analysis consists of two parts: the cointegration analysis to infer the functional network and prediction of the accuracy of the response from pre-stimulus EEG activity.

### 3.1. Fitting the cointegration model

The dataset of each participant was stratified into six subsets, and for each of them, one network was estimated by the cointegration analysis. The split was done according to the response accuracy (two categories: correct and incorrect) and the period within the experimental trial (three categories: fixation, stimulus and masking period). The fixation period was a 500 ms time window prior to the stimulus onset; the stimulation period was between 20 and 110 ms time window, and the masking period was the 500 ms time window starting with the mask onset (figure 1*b*). The number of experimental trials with selected characteristics of the fitted models is presented in table 1.

**Table 1.** The number of observations *N* in the datasets after splitting the data according to the accuracy of the answer and the stage of the visual task, the cointegration ranks ${\hat{r}}_{RSC}$ estimated by the rank selection criterion and the tuning parameters *γ* and *ω* used in model fitting.

participant	accuracy	no. of trials	period		
			fixation	stimulus	masking
1	correct	416	*N* = 53 248	*N* = 7, 305	*N* = 52 832
			${\hat{r}}_{RSC}=25$	${\hat{r}}_{RSC}=14$	${\hat{r}}_{RSC}=22$
			*γ* = 2 · 10^−3^	*γ* = 1 · 10^−2^	*γ* = 2 · 10^−3^
			*ω* = 0.75	*ω* = 0.75	*ω* = 0.5
	incorrect	193	*N* = 24, 704	*N* = 1, 815	*N* = 24, 511
			${\hat{r}}_{RSC}=23$	${\hat{r}}_{RSC}=11$	${\hat{r}}_{RSC}=19$
			*γ* = 3 · 10^−3^	*γ* = 1 · 10^−2^	*γ* = 3 · 10^−3^
			*ω* = 0.75	*ω* = 0.25	*ω* = 0.75
2	correct	414	*N* = 52, 992	*N* = 7, 745	*N* = 52 578
			${\hat{r}}_{RSC}=20$	${\hat{r}}_{RSC}=12$	${\hat{r}}_{RSC}=17$
			*γ* = 2 · 10^−3^	*γ* = 4 · 10^−2^	*γ* = 4 · 10^−3^
			*ω* = 1	*ω* = 0.25	*ω* = 0.5
	incorrect	173	*N* = 22, 144	*N* = 1, 468	*N* = 21 971
			${\hat{r}}_{RSC}=18$	${\hat{r}}_{RSC}=9$	${\hat{r}}_{RSC}=15$
			*γ* = 6 · 10^−3^	*γ* = 4 · 10^−2^	*γ* = 3 · 10^−3^
			*ω* = 0.5	*ω* = 0.75	*ω* = 0.75

*Cointegration rank.* The cointegration rank was investigated through the criteria described in §2.2.5. The likelihood ratio tests using bootstrap were too time consuming and therefore not performed. We focused on the fixation period, where the process is expected to be most stable. We then used the same rank for stimulation and masking periods.

**Figure 2.**
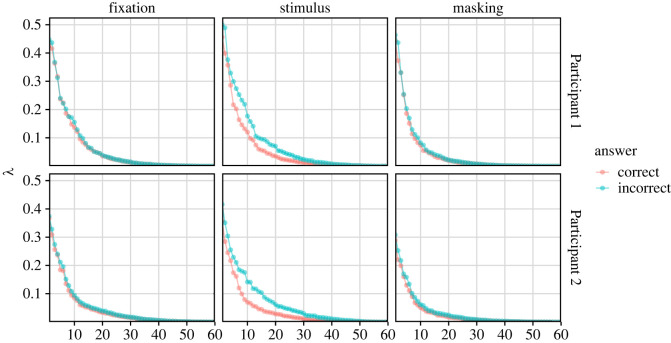
Ordered eigenvalues of equation (2.18) for the six subsets of the data.

The visual assessment of the scree plots of the eigenvalues (figure 2) reveals that the most rapid decrease is observed for ranks between 0 and 20, consistently across all six studied scenarios. The stimulation period has in general larger eigenvalues than the other periods, and the data from trials with incorrect answers have larger eigenvalues than the data from trials with a correct answer in the stimulation period. This is probably due to smaller sample sizes and not evidence of a change in the cointegration rank. The eigenvalues for the masking period decrease to zero earlier than for the fixation period. This could indicate that the activity in the masking period is less cointegrated and driven by more stochastic trends than in the fixation period, since the amount of data is similar.

**Figure 3.**
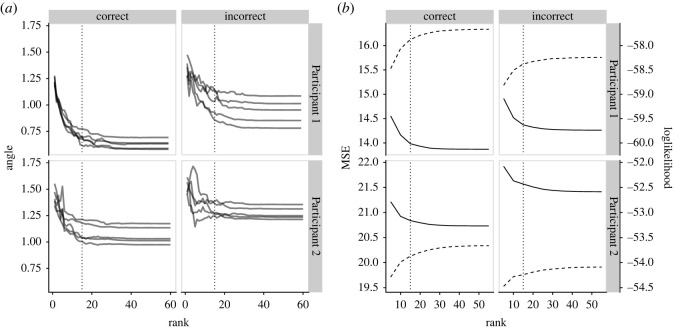
(*a*) The angles $\Theta ({\hat{\Pi }}_{r}^{\left(k\right)},{\hat{\Pi }}_{p}^{\left(-k\right)})$ between ${\hat{\Pi }}_{r}^{\left(k\right)}$ (the estimate of Π under presumed rank *r* fitted to fold *k* = 1, …, 5) and ${\hat{\Pi }}_{p}^{\left(-k\right)}$ (the estimates of Π under presumed full rank and when excluding data from fold *k*). (*b*) The cross-validated mean squared error (solid) and log-likelihood (dashed). The plots depict the fixation period only. The vertical dotted line marks the selected rank *r* = 15.

The angles $\Theta ({\hat{\Pi }}_{r}^{\left(k\right)},{\hat{\Pi }}_{p}^{\left(-k\right)})$ after splitting data into five folds (figure 3) are nearly constant for ranks larger than 15, and the variability of $\Theta ({\hat{\Pi }}_{r}^{\left(k\right)},{\hat{\Pi }}_{p}^{\left(-k\right)})$ across folds *k* is also higher for those ranks. The MSE of prediction and the average cross-validated log-likelihood were calculated for ranks *r* = 5, 10, …, 55. MSEs attain minimum for *r* = 55, except when the answer of Participant 2 is incorrect, in which case the minimum is achieved for *r* = 50. The cross-validated likelihood always attains its maximum for *r* = 55. However, both criteria change only little for *r* > 15. Finally, ranks estimated by the rank selection criterion (table 1) were between 9 and 25. We continue the analysis with *r* = 15 in all set-ups.

The models were fitted by the Johansen procedure with elastic net penalization on *α*. In the following, we comment on the main results for Participant 1. For the complete results of both participants, see the electronic supplementary material.

**Figure 4.**
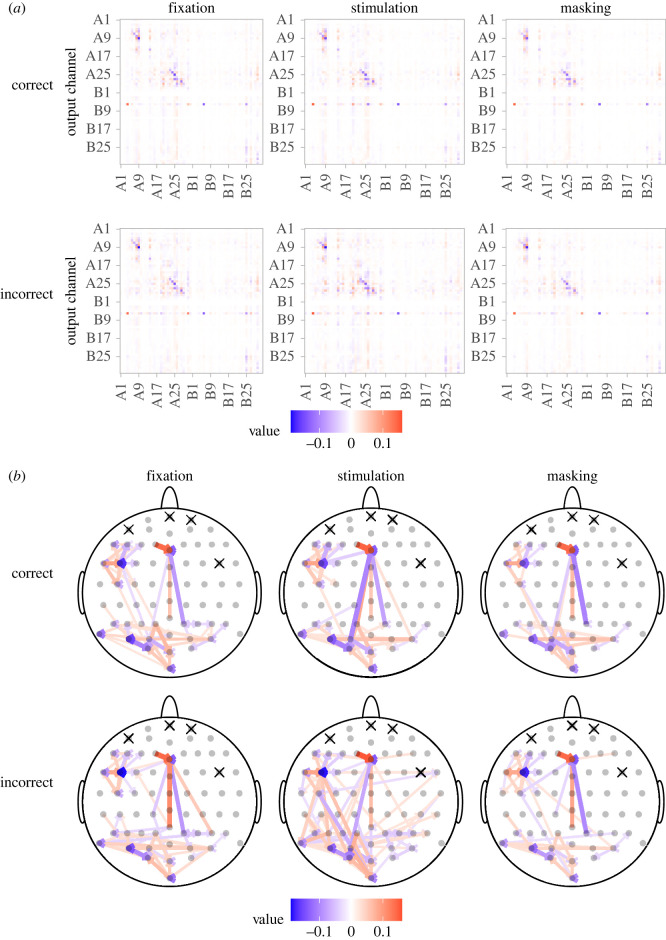
Estimated network for Participant 1. (*a*) Estimates of Π and (*b*) the induced networks for the six categories of the data. The crossed-out channels were excluded from the model.

*Network.* Two main patterns are visible in $\hat{\Pi }$ (figure 4*a*). First, the row for channel B6 stands out with many large elements, indicating that B6 is affected strongly by other channels. Second, there are a few regions with larger positive or negative elements, signalling clusters of interlinked activity. One such cluster consists of channels around channel A9, and another cluster consists of channels A20 to A30. This is also shown in figure 4*b*, which shows strong input into B6 and lots of connections in the left frontal region and in the occipital lobe.

*Test of difference between networks for correct and incorrect trials*. By mere eye, there seems to be little difference between the estimated networks. Therefore, we compared the cointegration model fitted separately to correct and incorrect trials with the model fitted to all trials, separately for the periods of fixation, stimulation and masking. The results for both participants (table 2) indicate a highly significant difference between the cointegration models for correct and incorrect trials in all three periods.

**Table 2.** Likelihood ratio tests of structural differences between trials with correct and incorrect answers.

participant	period	*Q*(*H*_0_|*H*_*a*_)	d.f.	$\chi_{Df}^{2}(0.95)$	*p*-value
1	fixation	15593.4	1604	1698.3	<0.001
	stimulation	5728.7	1604	1698.3	<0.001
	masking	15124.3	1604	1698.3	<0.001
2	fixation	11205.2	1635	1730.2	<0.001
	stimulation	4579.9	1635	1730.2	<0.001
	masking	11632.8	1635	1730.2	<0.001

Since the cointegration models differ visually little for the six subsets of data, in the following, we will comment on $\hat{\beta }$, $\hat{\alpha }$ and $\hat{\mu }$ only for the fixation period in trials with a correct answer to illustrate the main features. The estimates for the remaining five subsets can be found in the supplementary material. It appears that the main pattern is specific to the participant, since the cointegration models for participant 2 are clearly different from participant 1, but do show very similar features within participant across the six subsets of data.

**Figure 5.**
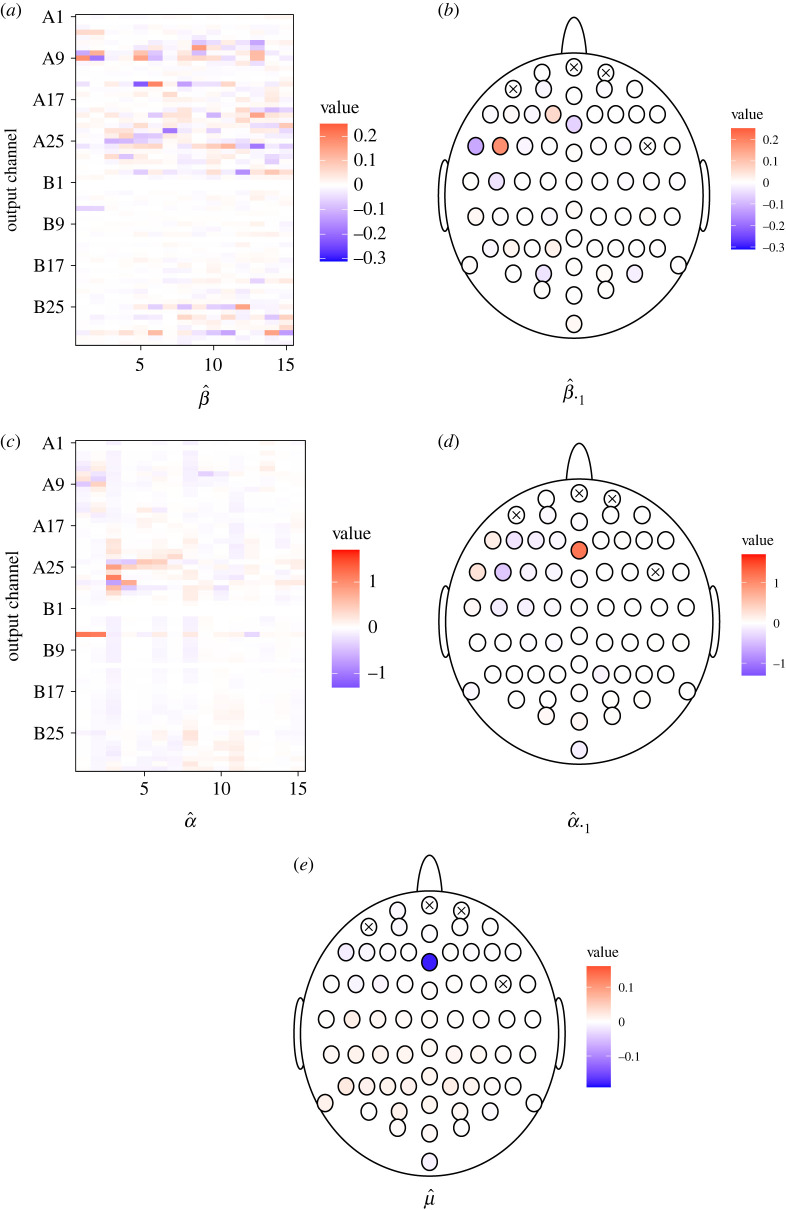
Fitted values for Participant 1. (*a*) Estimate of *β*, (*b*) weights of the first cointegration vector, (*c*) estimate of *α*, (*d*) loadings corresponding to the first cointegration relationship and (*e*) estimate of the drift *μ* for the fixation period in trials with a correct answer. The crossed-out channels were excluded from the analysis.

*Cointegration vectors.* Each of the 15 columns of $\hat{\beta }$ (figure 5*a*) represents weights of one cointegration vector. They have almost zero weights from channels B7 to B20. This indicates that these channels play a minor role in the joint dynamics of the system.

Figure 5*b* shows the first cointegration vector and how the channels with largest weights are located on the scalp. The most distinctive channels are two neighbouring channels A8 and A9 in the left frontal region and two neighbouring channels A4 and B6 in the centre of the frontal lobe. Neighbouring channels have weights of similar absolute value, but opposite signs, which indicates that they tend to have the same level of activity at equilibrium. This may result naturally from the presence of a strong connection between the neighbouring channels due to volume conduction [3]. However, there could be another mediator channel as well.

*Loadings.* It is important to note that $\hat{\alpha }$ can only be interpreted in the light of the accompanying matrix $\hat{\beta }$, which is not uniquely identifiable. For the estimate $\hat{\beta }$ satisfying the normalization condition (2.9), then larger absolute values of *α* (figure 5*c*) appear only in channel B6, around A9 and A20–A30, so the equilibrium cointegrated state specified by columns of $\hat{\beta }$ that satisfies (2.9) is achieved primarily by adjustments of these channels. For example, the equilibrium in the first cointegration vector (figure 5*d*) is achieved through adjustments of channel B6, which has the most significant loading in the first column of $\hat{\alpha }$. The sign of the loading has no biological meaning because it depends on the particular form of the cointegration vector, where signs of the weights could be reversed.

The channels having a noticeable non-zero weight in the cointegration vector usually also have a noticeable non-zero loading. Thus, if the equilibrium state is not temporarily met, the channels defining the equilibrium are also subject to the largest adjustments. However, this does not apply to channel A4, which plays a large role in the cointegration relationship, but has a loading close to zero. Thus, this channel’s activity evolves independently of whether the cointegration equilibrium is achieved, while the remaining channels adjust their activity to match the activity of A4 required in the cointegrated state.

*Drift.* For EEG data, we do not expect large drifts. First, the range of EEG signals is limited by natural laws. Second, forcing the signals to sum to zero limits possible drifts. This is shown in figure 5*e*. The majority of channels have drifts close to zero. Only channel B6 stands out with a significant negative drift.

### 3.2. Brain networks identified as predictors of performance in the visual task

In this subsection, we investigate whether the brain states identified by the cointegration analysis for the period right before the stimulus onset are predictive of whether a participant can identify the visual stimulus correctly. We fit a logistic model, where the response is the accuracy of the answer. One predictor is then the goodness of fit of two alternative cointegration models.

We construct the variable *d*_*m*_ for trial *m* ∈ {1, …, *M*}, which quantifies how well data from the *m*th trial, *x*^(*m*)^, are fit by a model *A* with parameters $\left(\mu_{A},\Pi_{A},\Sigma_{A}\right)$ compared with a model *B* with parameters $\left(\mu_{B},\Pi_{B},\Sigma_{B}\right)$. We capture it by the difference in the log-likelihoods under the two models [33],

3.1 $$d_{m}=\log L(\mu_{A},\Pi_{A},\Sigma_{A};x^{\left(m\right)})-\log L(\mu_{B},\Pi_{B},\Sigma_{B};x^{\left(m\right)}).$$

Model *A* is the cointegration model (2.3) fitted to data in the 100 ms time window prior to the stimulus onset in the trials with a correct answer, whereas Model *B* is with an incorrect answer. We used 100 ms to capture the network right before the stimulus onset and simultaneously to allow for a full period of 10 Hz oscillatory activity, which has previously been suggested to drive the predictive value in the pre-stimulus EEG [17]. We applied a leave-one-trial-out principle, so if the answer in trial *k* was correct, the data from this trial were excluded for fitting Model *A*, and if the answer was incorrect, the data were left out when fitting Model *B*.

Furthermore, the following covariates were included to control for the experimental conditions:

— *Stimulus duration* (*sd*_*m*_), with four levels: {20, 40, 70, 110 ms}.
— *Stimulus orientation* (*o*_*m*_), with eight levels: {0°, 45°, 90°, …, 315°}.
— *Luminance contrast* (*c*_*m*_), with two levels: {high, low}.
— *Fixation duration* (*fd*_*m*_), with 11 levels: {1.5, 1.6, …, 2.5 s}.
— *Stimulus location* (*loc*_*m*_), with two levels: {left, right}.

All the covariates, except for *d*_*m*_, are treated as categorical variables, even though some of them could be naturally treated as continuous variables. We do so to capture possible nonlinear effects and to explain the maximum portion of variability.

We considered a range of logistic models. The full model with all variables included (*M*_1_) has the form

3.2 $$\log\left(\frac{\;p_{m}}{1-p_{m}}\right)=\alpha +\beta_{sd_{m}}+\beta_{o_{m}}+\beta_{d}d_{m}+\beta_{c_{m}}+\beta_{\;fd_{m}}+\beta_{loc_{m}},$$

where *p*_*m*_ is the probability of a correct answer in trial *m*. This model was compared with Model *M*_2_, where the log-likelihood difference was excluded from the set of predictors. Moreover, we fitted six single-predictor models *M*_01_−*M*_06_ with only one variable at a time, to assess the predictive power of each covariate on their own.

**Figure 6.**
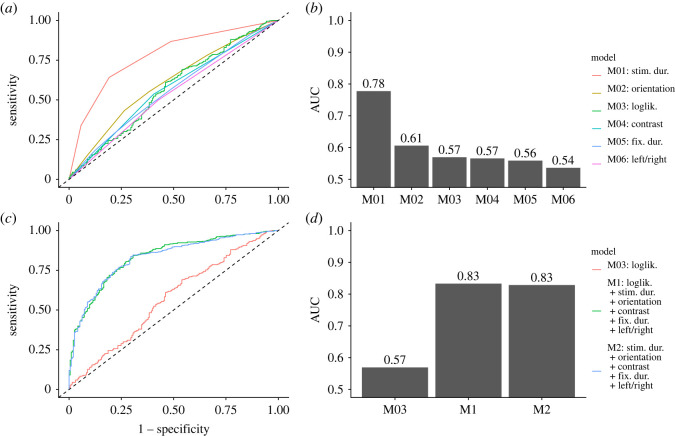
The ability of logistic models (*a*,*b*: *M*_01_–*M*_06_, *c*,*d*: *M*_1_, *M*_2_ and *M*_03_) to predict correct answers of Participant 1 captured by ROC curves (*a*,*c*) and the corresponding areas under the curve AUC (*b*,*d*).

The fit of the models and their ability to predict the accuracy of the visual identification is quantified with Akaike information criteria (AIC), Bayesian information criteria (BIC) and area under the curve (AUC) statistics in table 3, and receiver operating characteristic (ROC) curves corresponding to the models are shown in figure 6 for Participant 1. As expected, the *stimulus duration* is the variable with the highest predictive ability for both participants, when used as the only predictor (AUC = 0.78 for Participant 1 and AUC = 0.85 for Participant 2). It is followed by *stimulus orientation* (AUC=0.61 and 0.63, respectively) and *log-likelihood difference* (AUC = 0.60 and 0.61, respectively). The predictive ability of *luminance contrast*, *stimulus location* and *fixation duration* is weaker. These predictors are also borderline significant (the contrast is significant for Participant 1 and insignificant for Participant 2; the stimulus location is insignificant for Participant 1, but significant for Participant 2) or even insignificant (fixation duration for both participants) in the one-predictor models *M*_04_, *M*_05_ and *M*_06_ (compared with table 4).

**Table 3.** Fitting and prediction performance of the logistic models.

model	covariates	d.f.	Participant 1			Participant 2		
			AIC	BIC	AUC	AIC	BIC	AUC
*M*_01_	stim. duration	3	627.0	644.6	0.78	481.6	499.1	0.85
*M*_02_	stim. orientation	7	757.4	792.7	0.61	703.3	738.3	0.63
*M*_03_	loglik. diff.	1	741.2	750.0	0.60	700.9	709.7	0.61
*M*_04_	contrast	1	755.4	764.3	0.57	712.2	721.0	0.54
*M*_05_	fix. duration	10	776.3	824.8	0.56	724.2	772.3	0.57
*M*_06_	left/right	1	761.9	770.7	0.54	709.1	717.9	0.56
*M*_1_	all	23	598.8	704.7	0.84	439.8	544.8	0.91
*M*_2_	all except *d*_*m*_	22	616.6	718.1	0.83	448.6	549.2	0.91

**Table 4.** Significance tests of the covariates in the full model *M*_1_ and in the single covariate models *M*_01_ − *M*_06_. The reported numbers are *p*-values from drop-in-deviance tests.

covariate	Participant 1		Participant 2	
	*M*_1_	${\left\{M_{0i}\right\}}_{i=1}^{6}$	*M*_1_	${\left\{M_{0i}\right\}}_{i=1}^{6}$
stim. duration	<0.001	<0.001	<0.001	<0.001
stim. orientation	<0.001	0.007	<0.001	<0.001
loglik. difference	<0.001	<0.001	0.001	<0.001
contrast	<0.001	0.002	<0.001	0.058
fix. duration	0.915	0.784	0.628	0.470
left/right	0.020	0.094	0.008	0.010

Model 1 with all covariates has good predictive power (AUC = 0.84 for Participant 1, AUC = 0.91 for Participant 2). The predictive power of Model 2, where the log-likelihood difference is excluded, is nearly the same. However, the decrease of fit is statistically significant both for Participant 1 (*p* < 10^−4^) and Participant 2 (*p* = 0.002). This suggests that the network right before the stimulus onset impacts the performance in the visual task. Nevertheless, data from more participants would be needed to confirm that the effect of the brain state before the stimulus onset on the cognitive performance is general.

## 4. Discussion

We have shown how cointegration methodology can be applied to infer functional networks in EEG data and have thereby expanded the available statistical toolbox for infering functional connectivity. We have applied the cointegration analysis on EEG data obtained from a visual task experiment with two participants.

Cointegration analysis is based on a VAR model, which is not new in EEG data analysis [7–9]. Our use of VAR models differs in two ways. First, a standard VAR model has no restriction on the rank of Π, which means that $\hat{\Pi }$ has full rank almost surely and no stochastic trends are allowed. If the data are non-stationary, the fitted model may have no link to existing relations between EEG channels due to the phenomenon of spurious regression [10]. On the contrary, the cointegration approach starts with estimating the rank of Π; therefore, if non-stationarity is present, it is taken into account correctly.

Second, the VAR model in our approach is not just an auxiliary intermediate step as in other procedures of network recovery, but the actual model of interactions between EEG units. The parameters of the rank-restricted VAR model fitted as part of the cointegration analysis can also be used in further analysis as illustrated earlier or e.g. used to deduce Granger causality. We only use a VAR model of order 1, since a higher order VAR model of high dimension would have too many parameters to be fit reliably. Furthermore, we did not achieve a substantially better fit by adding more lags (results not shown). A VAR(1) model has moreover the advantage of a direct link to the continuous time Ornstein–Uhlenbeck model, which has a straightforward interpretation. Nevertheless, it should always be checked if a VAR model with lag order 1 is sufficient to fit a particular dataset, e.g. by inspecting the autocorrelation function of the residuals. We did not find any substantial autocorrelation in the residuals from most channels, and adding more lags did practically not change the residuals (results not shown).

We found that the cointegration analysis produced a distinct subject-specific network that did not differ visually between experimental conditions (figure 4), but nevertheless was predictive of task performance. Specifically, the pre-stimulus network predicted the response accuracy on the subsequent visual identification task. This finding corroborates previous studies investigating the effect of specific spontaneous and oscillatory brain activity features prior to the onset of a sensory stimulus on identification performance [17]. Thus, the cointegration analysis offers an exploratory approach to understand if and how the brain state at one instance in time affects behavioural performance in a subsequent task.

The classification of brain states employs a logistic model, but other approaches to brain state classification exist, such as neural networks [9] or quadratic discriminant analysis [34]. However, to evaluate the specific contribution of the brain state compared with other covariates, a parametric approach such as logistic regression is more suitable.

We estimated the functional connectivity network between channels. However, the measured potentials are only indirect indicators of unknown sources of neuronal activity. The underlying sources can influence measured activity at several nearby channels and lead to spurious correlations between EEG channels. This problem could be potentially fixed by applying some of the algorithms of EEG source localization [35] and inferring the functional connectivity network between the reconstructed sources [36,37]. The reliability of the cointegration methodology with the reconstructed sources needs to be further investigated. Altogether, the network inferred from EEG channels should be interpreted cautiously. However, if changes in connectivity caused by an experimental manipulation are of main interest, as in the logistic model for predicting performance in the visual task, the ambiguity in the connectivity of the true sources is not an issue.

## Data Availability

The EEG data from the visual task experiment, the full data analysis for both participants and the underlying R code are publicly available at the online repository https://doi.org/10.17894/ucph.f19b3ddd-ea40-4d96-8787-c41aac9bd2e7 [38].
